# Estradiol and hormone therapy: Associations with cognitive and behavioural changes in postmenopausal females

**DOI:** 10.1002/alz70857_102190

**Published:** 2025-12-24

**Authors:** Jasper F.E.U. Crockford, Dylan X. Guan, Maryam Ghahremani, Gillian Einstein, Cindy K Barha, Eric E. Smith, Zahinoor Ismail

**Affiliations:** ^1^ Hotchkiss Brain Institute, University of Calgary, Calgary, AB, Canada; ^2^ University of Toronto, Toronto, ON, Canada; ^3^ Rotman Research Institute, Toronto, ON, Canada; ^4^ Hotchkiss Brain Institute, Calgary, AB, Canada; ^5^ University of Calgary, Calgary, AB, Canada; ^6^ Department of Clinical Neurosciences, University of Calgary, Calgary, AB, Canada

## Abstract

**Background:**

Estradiol is an ovarian hormone present during reproductive years. During menopause, estradiol decline may trigger or exacerbate pathological mechanisms of AD. Estrogen‐based hormone therapies (HT), may mitigate cognitive decline and AD, although findings are inconsistent. Alongside later‐life emergent and persistent cognitive changes, later‐life emergent and persistent behavioural and personality changes (i.e., mild behavioral impairment ‐ MBI), is an early marker of dementia, but its relationship with estradiol is unknown. This study examined relationships between postmenopausal estradiol levels, HT‐use, and cognitive and behavioural symptoms.

**Method:**

Participant data were from the Comprehensive Assessment of Neurodegeneration and Dementia study, including 333 postmenopausal females (*n* = 97 used HT) spanning the neurocognitive spectrum. Estradiol levels were measured from blood plasma samples. Cognition was assessed using the Montreal Cognitive Assessment (MoCA); scores ≥26 were considered normal cognition. MBI symptoms were measured with the Mild Behavioral Impairment Checklist (MBI‐C); higher scores denoted greater symptom severity. Associations between estradiol levels and MoCA and MBI‐C scores were analyzed using linear and negative‐binomial regressions, respectively, including an estradiol*HT‐use interaction term. All models were adjusted for age, education, race, HT‐use, age at menopause onset, and type of menopause (spontaneous, oophorectomy, hysterectomy, other reasons).

**Result:**

Participants (mean age 71.3±6.8 years) included 27.0% cognitively normal, 23.7% with subjective cognitive decline, 34.5% with mild cognitive impairment, and 14.7% with dementia. Neurocognitive status did not predict estradiol levels. Estradiol levels were not associated with MoCA score (b=0.29, 95%CI [‐1.00, 0.42], *p* = 0.66) and the relationship was not modified by HT‐use (b_int_=1.41, 95%CI [‐0.11, 2.93], *p* = 0.16). HT‐use was not independently associated with MoCA score (b=0.79, 95%CI [‐0.24, 1.83], *p* = 0.2). However, higher estradiol levels were associated with lower (i.e., better) MBI‐C scores (b=0.51, 95%CI [0.28, 0.94], *p* = 0.05), independent of HT‐use (b_int_=0.96, 95%CI [0.22, 4.05], *p* = 0.95). Notably, HT‐use was independently associated with lower MBI‐C scores (b=0.24, 95%CI [0.11, 0.55], *p* <0.01).

**Conclusion:**

Higher estradiol levels and HT‐use were independently associated with lower MBI symptom severity but not better MoCA scores, suggesting neuropsychiatric markers such as MBI may be more sensitive to postmenopausal estradiol changes than cognitive assessments. However, longitudinal biomarker studies are needed to elucidate underlying mechanisms.

